# In vivo knockdown of intersectin-1s alters endothelial cell phenotype and causes microvascular remodeling in the mouse lungs

**DOI:** 10.1007/s10495-012-0762-x

**Published:** 2012-10-07

**Authors:** Cristina Bardita, Dan Predescu, Matthew J. Justice, Irina Petrache, Sanda Predescu

**Affiliations:** 1 Department of Pharmacology, Rush University, 1735 W. Harrison St., Chicago, IL 60612 USA; 2 Department of Medicine, Indiana University, Indianapolis, IN USA

**Keywords:** Apoptosis, Endothelial cells, Microvascular remodeling, Phenotypic changes, siRNA

## Abstract

Intersectin-1s (ITSN-1s) is a general endocytic protein involved in regulating lung vascular permeability and endothelial cells (ECs) survival, via MEK/Erk1/2^MAPK^ signaling. To investigate the in vivo effects of ITSN-1s deficiency and the resulting ECs apoptosis on pulmonary vasculature and lung homeostasis, we used an ITSN-1s knocked-down (KD_ITSN_) mouse generated by repeated delivery of a specific siRNA targeting ITSN-1 gene (siRNA_ITSN_). Biochemical and histological analyses as well as electron microscopy (EM) revealed that acute KD_ITSN_ [3-days (3d) post-siRNA_ITSN_ treatment] inhibited Erk1/2^MAPK^ pro-survival signaling, causing significant ECs apoptosis and lung injury; at 10d of KD_ITSN_, caspase-3 activation was at peak, terminal deoxynucleotidyl transferase dUTP nick-end labeling (TUNEL)-positive ECs showed 3.4-fold increase, the mean linear intercept (MLI) showed 48 % augment and pulmonary microvessel density as revealed by aquaporin-1 staining (AQP-1) decreased by 30 %, all compared to controls; pulmonary function was altered. Concomitantly, expression of several growth factors known to activate Erk1/2^MAPK^ and suppress Bad pro-apoptotic activity increased. KD_ITSN_ altered Smads activity, downstream of the transforming growth factor beta-receptor-1 (TβR1), as shown by subcellular fractionation and immunoblot analyses. Moreover, 24d post-siRNA_ITSN_, surviving ECs became hyper-proliferative and apoptotic-resistant against ITSN-1s deficiency, as demonstrated by EM imaging, 5-bromo-deoxyuridine (BrdU) incorporation and Bad-Ser^112/155^ phosphorylation, respectively, leading to increased microvessel density and repair of the injured lungs, as well as matrix deposition. In sum, ECs endocytic dysfunction and apoptotic death caused by KD_ITSN_ contribute to the initial lung injury and microvascular loss, followed by endothelial phenotypic changes and microvascular remodeling in the remaining murine pulmonary microvascular bed.

**Electronic supplementary material:**

The online version of this article (doi:10.1007/s10495-012-0762-x) contains supplementary material, which is available to authorized users.

## Introduction

Intersectin-1s (ITSN-1s) is a member of ITSN family of proteins, best-known for its critical role in coordinating distinct steps in clathrin-coated vesicles (CCVs) and caveolae endocytosis, [1–4]. Due to its endocytic function and multimodular structure, ITSN-1s links growth factors receptors and regulatory proteins predominantly localized in CCVs and caveolae, to signaling networks [1, 2, 5, 6]. ITSN-1s activates Ras, via its association with mSOS, a GEF for Ras, leading to downstream Erk1/2^MAPK^ signaling activity [7]. Previous studies indicated that downregulation of ITSN-1s via siRNA inhibits Erk1/2^MAPK^ and its direct activator MEK, leading to mitochondrial apoptosis of cultured human lung microvascular ECs [8]. The findings are consistent with pro-survival signaling orchestrated by ITSN-1s via mSOS/Ras/Erk1/2^MAPK^ pathway, known to play a pivotal role in cell survival, proliferation and vascular remodeling, [9–11].

ECs dysfunction and apoptotic death are common events in many pathological conditions such as sepsis, inflammatory syndromes, atherosclerosis, and pulmonary arterial hypertension (PAH), [12]. Recent studies showed that increased pulmonary ECs apoptosis caused by loss-of-function mutations in bone morphogenetic protein receptor-2 (BMPR2) may represent a possible initiating mechanism in PAH, [13]. Moreover, studies using chronically hypoxic SU5416-treated rats indicated that increased apoptosis of ECs created conditions favoring the emergence of apoptosis-resistant cells and that blockade of ECs growth factors receptors worsen the pathological vascular remodeling [14].

ITSN-1s is one of the recently identified substrates for the cytotoxic protease granzyme B (GrB), [15], raising the possibility that during inflammatory processes, major pathogenic components of pulmonary vascular remodeling, [16], ITSN-1s can be cleaved and thus, expression of full-length protein reduced. Recently, we have shown that ITSN-1s deficiency is relevant to the pro-inflammatory ECs dysfunction induced by lipopolysaccharide; the decrease in ITSN-1s mRNA and protein expression were countered by Bcl-X_L_ and survivin upregulation, as well as Bim downregulation, events thought to protect ECs from impending apoptosis [17]. Little is known about ITSN-1s deficiency and its implications in human lung diseases, since no studies have examined the consequences of KD_ITSN_ and the subsequent ECs dysfunction and apoptotic death in vivo. With the recent introduction of siRNA, the silencing of genes involved in pathologic mechanisms is carried out extensively in vitro, but more limited in vivo, due to either inefficient KD or adverse effects, such as immune responses, toxicity or off-target effects [18]. Recently, using cationic liposomes/siRNA_ITSN_ complexes delivered retro-orbitally into mice we knocked down ITSN-1s protein and mRNA levels in mouse lungs for 24 consecutive days [19]. The amounts of siRNA_ITSN_ injected (100 μg siRNA/mouse) were significantly lower compared to previously reported data [20], and sufficient for efficacy with no detectable adverse effects. Using this KD_ITSN_ mouse model, we have shown that lung ECs deficient of ITSN-1s, while impaired in caveolae and CCVs endocytosis, show up-regulation of alternative endocytic pathways (i.e. enlarged endocytic structures, membranous rings and tubules, etc.,) to compensate for deficient vesicular trafficking [19]. It is well-known that endocytosis and vesicular trafficking have significant impact on growth factors receptors signaling/activity and their associated proteins and downstream targets [21–23]. Endocytic dysfunction and altered intracellular trafficking and signaling of cell surface receptors such as TβRs, BMPR2 and tyrosine kinase receptors [i.e. vascular endothelial growth factor receptor (VEGF-R), platelet-derived growth factor receptor, (PDGF-R), insulin receptor (IR) etc.] have been implicated in the pathogenesis of PAH, emphysema and chronic obstructive pulmonary disease [24–27], pointing to implications of disrupted intracellular membrane trafficking in the pathobiology of vascular remodeling. However, studies on the potential involvement of ECs dysfunctional endocytic membrane traffic and the consequences on growth factor(s) receptors signaling in the pathology of lung microvascular remodeling are limited. Thus, the aim of this study was to elucidate the in vivo effects of ECs apoptosis and impaired endocytic membrane trafficking caused by ITSN-1s deficiency on mouse lung vasculature and lung homeostasis, using the lung-specific KD_ITSN_ mouse model we generated [19]. Here, we show that specific downregulation of ITSN-1s expression in mouse lungs causes ECs death and lung injury, followed by endothelial phenotypic changes toward hyper-proliferation and apoptosis-resistance, leading to lung repair and microvascular remodeling in the remaining pulmonary microvascular bed. Overall these findings established an important role of ITSN-1s in the ECs function and maintenance of lung homeostasis, while illustrating the effectiveness of siRNA in vivo.

## Materials and methods

### Delivery of siRNA_ITSN_ to mouse lungs

Young mice (6–8 weeks), as well older mice (24 weeks) were used in the studies. A specific ITSN-1s siRNA sequence (100 μg siRNA/mouse) targeting ITSN-1 gene was delivered via cationic liposomes, by retro-orbital injection, into mouse lungs as in [19, 28]. Pilot studies we performed to determine the siRNA_ITSN_ amount needed for efficient KD_ITSN_ without structural alteration of mouse organs, lung included. Chronic inhibition of ITSN-1s, was achieved by repeated siRNA_ITSN_/liposomes delivery, every 72 h, for 24d as in [19]. Mice were sacrificed at 3, 10 15 and 24d; Three to four mice per experimental condition [controls (wt-mice, vehicle- and non-specific siRNA-treated mice) and siRNA_ITSN-_treated mice] were used; all these experiments were repeated three times. No mouse mortality was recorded during the 24d of this experimental approach. All animal studies were performed in accordance with the guidelines of the University Institutional Animal Care and Use Committee. All surgeries were performed under anesthesia using i.p. delivery of 1 ml/kg body weight of ketamine hydrochloride/xylazine hydrochloride solution, (Sigma, St. Louis, MO).

### Protein extraction

Lungs free of blood by in situ perfusion (1.5 ml/min warm Hank’s solution) were homogenized in 150 mM NaCl, 50 mM Tris, pH 8.0 and protease inhibitors; total lysates were prepared by adding NP-40, to a final concentration of 1.0 %, for 2 h, at 4 °C. The ensuing lysates were clarified by centrifugation in a Beckman ultracentrifuge with a TLA-55 rotor, at 45,000 rpm, for 45 min, at 4 °C. Protein concentration was determined by the microBCA, (Pierce Biotechnology, Rockford, IL), with a bovine serum albumin (BSA) standard.

### Subcellular fractionation

Nuclear and cytosolic fractions of mouse lungs were prepared as in [29]. Lung tissue, free of blood by in situ perfusion of Hank’s solution, was washed twice with PBS and then transferred to hypotonic buffer (250 mM sucrose, 50 mM Tris–HCl, pH 7.4, 5 mM MgCl_2_, 1 mM DTT, and 1 mM phenylmethyl sulfonyl fluoride) for 30 min to allow swelling. The tissue was then Dounce-homogenized (20–30 strokes) with a tight-fitting pestle. The homogenate was centrifuged in a refrigerated Beckman benchtop centrifuge at 800×*g* for 15 min; the postnuclear supernatant served as cytosol, while the pellet, which contains the nuclei, was resuspended in a lysis buffer and centrifuged again as above. The supernatant served as the “nuclear” fraction and further used for assessing Smads nuclear translocation.

### Western blot and densitometry

Total protein (70 μg/lane) of mouse lung lysates was subjected to 4–20 % SDS PAGE, transferred to nitrocellulose membranes, followed by Western blot as in [30]. The antibodies (Abs)—AQP-1, Smad4/7 pAbs, histone-3, VEGF-A, a dual BMP-2/4 mAbs and phospho-Smad2 pAb, (Santa Cruz Biotechnology, Santa Cruz, CA), Bad and phospho-Bad pAbs, BrdU mAb, and phospho-Erk1/2 mAb, Erk1/2, TGFβ Smad1, phospho-Smad1/5/8, caspase-3 pAbs (Cell Signaling, Danvers, MA) phospho-Smad3 pAb (Abcam, Cambridge, MA) were used as recommended by the manufacturers, at dilutions established in preliminary experiments. ITSN-1s mAb (BD Biosciences, San Jose, CA) was used throughout the study to evaluate the efficient KD_ITSN_ in mouse lung tissue. The bands of immunoreactivity were visualized using appropriate horseradish peroxidase-conjugated Abs (eBioscience, San Diego, CA) and enhanced chemiluminescent substrate (Pierce, Rockford, IL). HyBlot CL films were subjected to densitometric analysis using ImageJ1.37v software.

### Lung histology

Controls and siRNA_ITSN_-treated mouse lungs were inflated with 1 % low-melting point agarose in 10 % formalin at a constant pressure of 25 cm H_2_O, allowing for homogenous expansion of lung parenchyma, and then fixed in 10 % paraformaldehyde for 48 h and paraffin-embedded [31, 32]. Thin sections (6 μm), were stained with hematoxylin/eosin (H&E) for lung tissue histological evaluation. Images (5 per lung section, 3 sections per mouse, 3–4 mice per group) were acquired with a 10 × lens and used for morphological assessment. MLI, a measure of interalveolar wall distance was evaluated on micrographs (35 micrographs randomly selected/specimen/3 different experiments) as in [31]. Briefly, the length of a line drawn across the lung section was divided by the total number of intercepts encountered in 100 lines per each section analyzed.

### Measurement of lung mechanics

Assessment of pulmonary lung function in controls and liposome/siRNA_ITSN_-treated mice was performed using the Flexivent system (Scireq, Montreal, Canada) as in [33, 34]. Briefly, mice were anesthetized with inhaled isoflurane in oxygen and orotracheally intubated with a 20-gauge intravenous cannula under direct vision. Isoflurane anesthesia was maintained throughout the measurements, and the mice were hyperventilated to eliminate spontaneous ventilation. Before testing each mouse, tube calibration was performed to remove the mechanical impedance of the tracheal cannula. Then, starting at functional residual capacity, the Flexivent was programmed to deliver seven inspiratory volume steps, for a total volume of 1 ml, followed by seven expiratory steps, pausing at each step for at least 1 s. Using this approach we evaluated the lung compliance (C_L_) and resistance (R_L_) as well as the inspiratory capacity (IC). Statistical analysis was performed with SigmaStat software using one-way ANOVA testing method (*n* = 4/each time point investigated). Statistical difference was accepted at *P* < 0.05. All data are presented as mean ± SEM.

### Lectin staining

Lectin staining was performed using biotinylated Griffonia Symplicifolia-1 (GS-1; Sigma) which binds specifically to α-galactosyl residues from microvascular endothelium [35]. After dewaxing and rehydration in PBS, the sections were blocked for unspecific binding with 1 % BSA in PBS, for 30 min. GS-1 (50 μg/ml) was applied for 1 h, at RT, in a humidified chamber. After washing, sections were incubated with NeutrAvidin dyelight (Sigma) for 30 min, and mounted with Prolong Antifade reagent (Molecular Probes).

### Aquaporin-1 (AQP-1) staining and morphometry

Deparaffinized and hydrated tissue sections were incubated for 15 min with 1.5 % H_2_O_2_ for blocking the endogenous peroxidase activity, followed by 1 h in 10 mM sodium citrate, pH 6.0, at 65 °C for antigen unmasking, as in [36]. Incubation with AQP-1 Ab for 30 min, at RT was followed by biotinylated universal Ab diluted in 2 % mouse serum and Vectastain ABC reagent (Vector Labs.) for 30 min, each. NovaRed substrate (Vector Labs.) was used for developing the red color. The number of microvessels labeled by AQP-1 was estimated by superimposing a rectangular grid over the sections and the microvessels included within the boundaries of each rectangle, were counted. The criterion used for identifying and counting microvessels was a circular/elliptical contour (<9 μm diameter) labeled by the AQP-1 Ab. When a capillary placed on the grid lines, it was considered only one time. Images (5 per lung section, 3 sections per mouse, and 3–4 mice per group) were acquired with a 63 × lens and used for morphological assessment. A minimum of 25 randomly chosen power fields per section form controls and ITSN-1s deficient mice were measured at a final magnification of 300×.

### Apoptosis and cell proliferation assays

TUNEL was carried out as per manufacturer’s instructions, (Roche, Indianapolis, IN). Paraffin-embedded sections from controls and siRNA_ITSN_-treated mouse lungs were deparaffinized, washed in PBS and treated with proteinase K, for 20 min at RT. The TUNEL mixture was applied for 60 min in a humidified atmosphere at 37 °C, in the dark. After washing, the sections were mounted using Prolong Antifade reagent. Lung sections incubated with TUNEL solution in the absence of TUNEL enzyme solution were used as negative control. TUNEL-positive ECs were identified by fluorescence microscopy using an excitation wavelength of 488 nm. Lung autofluorescence was reduced by incubation of lung sections, for 5 min with sulphorhodamin solution.

### BrdU incorporation

Mouse lungs ECs were labeled with BrdU solution (Roche, Indianapolis, IN) by intraperitoneal injection every 24 h, for two consecutive days. Paraffin-embedded sections were dewaxed and rehydrated in PBS for 10 min. Trypsin solution was applied for 5 min. The sections were denatured in 4 M HCl for 15 min and washed again in PBS. Next, each preparation was incubated with anti-BrdU/FITC Ab, for 45 min, at 37 °C in a humid chamber. After the last washing in PBS, the slides were mounted as above.

Quantification of BrdU- and TUNEL-positive nuclei was performed on medium-sized (50 ≥ diameter ≤ 100 μm) blood vessels. Using Zeiss Axiovision software we measured the diameter of the vessels by drawing an imaginary line perpendicular to the direction of the largest profile dimension which was then averaged with the value at its greatest width. A minimum of 25 vessels per section (3 sections per mouse, 3–4 mice per group) were used for counting the number of TUNEL- and BrdU-positive ECs nuclei. Data was normalized per 25 vessels.

BrdU-positive ECs were counted on 25 alveolar profiles in controls and siRNA_ITSN_-treated mouse lung sections. For treated specimens, only alveolar profiles with more than one ECs TUNEL-positive were considered on randomly chosen middle power fields.

### Collagen staining

Paraffin-embedded mouse lung sections were deparaffinized and hydrated in distilled water, stained in hematoxilyn and then subjected to Picrosirius Red (Polysciences) staining as instructed by the manufacturer. Finally, sections were dehydrated, cleared in xylene, and mounted with Paramount (Fischer Scientific). Picrosirius Red staining was imaged using a Zeiss Axio Observer Z1 Motorized Inverted Microscope; morphometry was performed using ImageJ1.37v software. Areas of interest were defined using 20–25 high power microscopic fields/mouse/experimental condition. The per area density of binding/staining was obtained by averaging the percentage values obtained for each area of interest analyzed.

### Statistical analysis

Statistical analysis was performed using Student’s two-tailed, unpaired *t* test for comparison between groups, for collagen staining and ANOVA testing for all other lung morphometry. Data are reported as mean ± SEM, Statistical significance was accepted at *P* < 0.05.

### EM

Mouse lungs, free of blood, were fixed by perfusion with 3 % paraformaldehyde and 1.5 % glutaraldehyde in 0.1 M Na cacodylate buffer, pH 7.3 [37]. Excised tissue was fixed in the same fixative mixture for one additional hour, at RT, post-fixed in Palade’s 1 % OsO_4_ on ice, and then stained with Kellenberger uranyl acetate. Selected specimens were dehydrated in graded ethanol and embedded in Epon812. Thin sections cut from blocks were mounted on nickel grids, stained with 2 % uranyl acetate and lead citrate, and finally examined and micrographed in a Joel 1220 TEM.

## Results

### Specific KD_ITSN_ in mouse lungs

ITSN-1s protein and mRNA levels were monitored at several time points after siRNA_ITSN_ delivery by Western blot and RT- and qPCR as in [19]. ITSN-1s protein and mRNA levels in siRNA_ITSN_-treated mouse lungs were about 75 % lower by reference to controls. Delivery of empty-liposomes or liposomes containing the non-specific siRNA did not affect the levels of ITSN-1s protein or mRNA, compared to controls. Comparable ITSN-1s downregulation, with the same timeline as in the lung, was detected in the brain, while in the heart, kidneys and liver was less efficient (not shown). This KD_ITSN_ murine model generated by liposome delivery of the siRNA_ITSN_ was used to further confirm in vivo that ITSN-1s deficiency induces ECs apoptosis in the pulmonary vasculature while affecting lung homeostasis.

### Acute KD_ITSN_ causes ECs apoptosis, microvessel loss and mouse lung injury

**Fig. 1:**
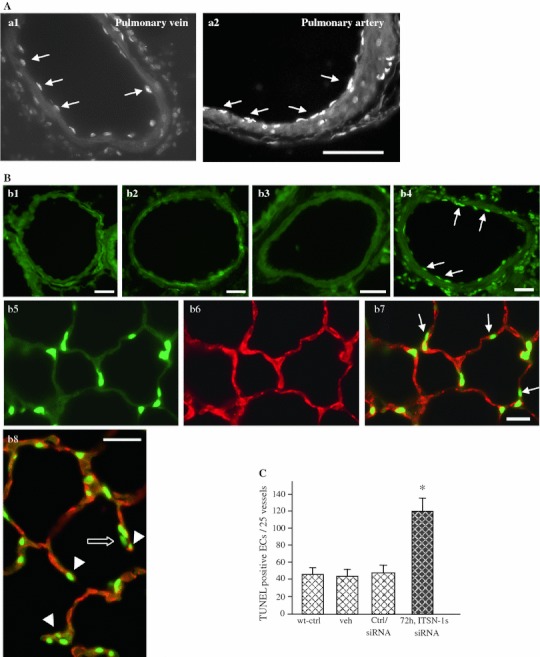
Acute inhibition (72 h post-siRNA_ITSN_) of ITSN-1s expression induces apoptosis in mouse lungs. **A**, **B** TUNEL demonstrates an increase in apoptotic ECs death in the lungs of mice with acute ITSN-1s inhibition (*a1*, *a2*, *b4*), by reference to controls, wild-type mice (*b1*), vehicle-injected mice (*b2*) or non-specific siRNA-treated mice (*b3*). TUNEL-positive ECs (*arrows*) were detected in large vessels—segments from a pulmonary vein (*a1*) and a pulmonary artery (*a2*) are shown, as well as in medium-sized (50–100 μm diameter) vessels (*b4*). *Bars* 30 μm (*a1*, *a2*), 20 μm (*b1*, *b2*). Lung sections of KD_ITSN_ mice (3d) were stained with TUNEL (*b5*) and CD31 Ab (*b6*) to evaluate the presence of apoptotic ECs within the microvessels of the alveolar septum (*b7*). *Arrows* in *b7*, indicate TUNEL-positive alveolar epithelial cells. The tips of damaged septal walls display TUNEL-positive ECs nuclei (*b8*, *arrowheads*) and alveolar epithelial cells nuclei (*b8*, *open arrow*) *Bars* 20 μm (*b3–b7*); 10 μm (*b8*). **C** Quantification of TUNEL-positive ECs in medium-sized vessels (50–100 μm) of controls and siRNA_ITSN_-treated mice lungs; *P < 0.05. Results are representative for three different experiments, with 3–4 mice/experimental condition

To investigate the effects of ITSN-1s deficiency on ECs apoptosis in the lungs of the KD_ITSN_ mouse we applied TUNEL reaction on paraffin-embedded sections of siRNA_ITSN_-treated mice, 72 h post-siRNA delivery. TUNEL-positive ECs, identified as elongated fluorescent puncta lining the blood vessel profiles were frequently associated with the perimeter of large vessels, (Fig. 1A, a1, a2), as well as medium-sized vessels, (Fig. 1B, b4). Morphometric analyses indicated an average of 4.6 TUNEL-positive ECs/mid-sized vessel profile in KD_ITSN_ mouse lung specimens, while in control specimens, wild-type mice, (Fig. 1B, b1), vehicle-treated (b2), or non-specific siRNA-treated mice (b3), their number decreased to 1.92 TUNEL-positive ECs/mid-sized vessel profile, 2.4-fold less compared to KD_ITSN_ specimens (Fig. 1C; Table 1). For specific evaluation of apoptotic ECs death within alveolar wall, TUNEL reaction (Fig. 1B, b5) was complemented by co-immunostaining for the generally accepted ECs marker, CD31 (CD31 Ab; Santa Cruz Biotechnology, Santa Cruz, CA; Fig. 1B, b6), as in [38]. Frequently, TUNEL-positive nuclei co-localized with cell membranes labeled by CD31/AlexaFluor594 Ab (Fig. 1B, b7, b8). TUNEL-positive cells were detected with a lesser frequency among alveolar epithelial cells and other resident cells of the lung (Fig. 1B, b7, arrows). Often, septal walls remnants displayed apoptotic ECs (Fig. 1B, b8, arrowheads) and epithelial cells (Fig. 1B, b8, open arrow) at their tip. TUNEL-positive lung cells were rare under control conditions (not shown).

**Table 1:** Morphometric analysis of ECs apoptosis, proliferation and microvessel density in the lungs of KD_ITSN_ mouse. The number of lung microvessels labeled by AQP-1 and GS-1 lectin was normalized per 30,000 μm^2^ surface area

Label/time point	Ctrl.	72 h	10d	24d
TUNEL + ECs/25 medium-sized vessels	48.0 ± 2.4	115 ± 2.9	165 ± 2.8	25 ± 2.0
AQP-1 microvessels, Ø < 9 μm	38.8 ± 4.0	28.81 ± 2.4	25.6 ± 4.0	ND
BrdU + ECs/25 medium-sized vessels	83.0 ± 5.0	ND	154 ± 3.8	173 ± 5.6
BrdU (1.5 × 102 μm alveolar wall length)	4.4 ± 1.0	ND	8.3 ± 1.5	9.4 ± 1.8
GS-1 lectin(microvessels, Ø < 20 μm)	365 ± 9.0	270 ± 4.5	ND	443 ± 7.1

**Fig. 2:**
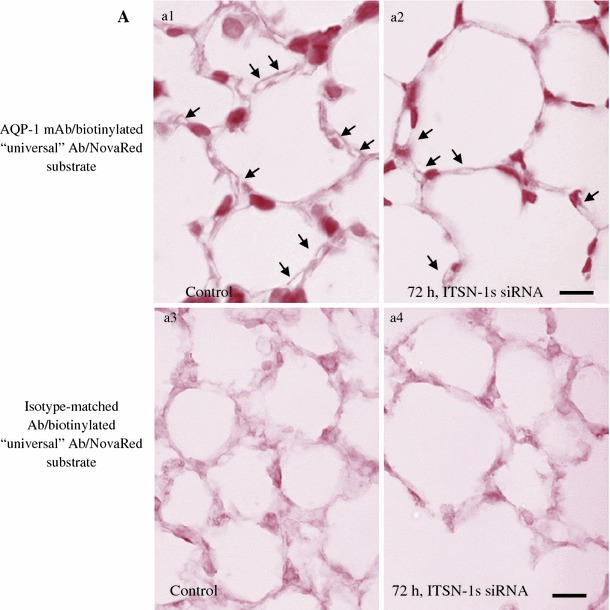
Acute inhibition (72 h post-siRNA_ITSN_) of ITSN-1s expression causes lung microvessel loss. **A** Micrographs of AQP-1staining of lung sections from controls (*a1*) and KD_ITSN_ mice, 3d, (*a2*), show decreased number of AQP-1 labeled microvessels (diameter or transverse diameter, respectively <9 μm) in siRNA_ITSN_ relative to control specimens. Arrows indicate microvessel profiles whose boundaries are labeled by AQP-1 Ab. Isotype-matched IgG staining of mouse lung sections of wt- (*a3*) and KD_ITSN_ mice (*a4*) produced no ECs staining. The NovaRed substrate produced a light-reddish background. *Bars* 10 μm (*a1*, *a2*), 15 μm (*a3*, *a4*). Results are representative for three different experiments, with 3–4 mice/experimental condition

Given the size of lung capillaries (<7–8 μm diameter), the thickness of the cellular and interstitial barrier in the thinnest regions of the alveolar septum (300–600 nm) [39], as well as the limited resolution of fluorescence microscopy in this range, accurate quantification of apoptotic ECs in lung microvessels was difficult. To obviate this limitation we monitored the changes in lung microvessels during acute KD_ITSN_ by AQP-1 immunostaining. AQP-1 water channels are expressed on apical and basolateral membranes of mammalian lung microvascular ECs and only occasionally in alveolar pneumocytes (type II), which are morphologically different from ECs and thus, not likely to interfere with identification of microvessels [40]. Numerous circular/elliptical profiles strongly labeled by AQP-1 Ab were detected in the lung microvasculature of wild-type mice (Fig. 2A, a1), contrasting with the limited number of similar AQP-1 outlined contours at 3d of siRNA_ITSN_ (Fig. 2A, a2). Note the reduced thickness of the septal wall, the decreased number of microvessels and the more diffuse AQP-1 staining in siRNA_ITSN_-treated lungs, most likely due to increased number of apoptotic events at this level. Quantitative measurements indicated that the mean number of lung microvessels per 30,000 μm^2^ surface area varies from 38.8 ± 4 (controls) to 28.81 ± 2.4 (3d). Omitting the primary Ab or using an isotype-matched IgG (Fig. 2A, a3, a4) show no ECs staining; a light-reddish background caused by the NovaRed substrate revealed thinner alveolar walls and evident signs of damage under siRNA_ITSN_ conditions (Fig. 2A, a4).

**Fig. 3:**
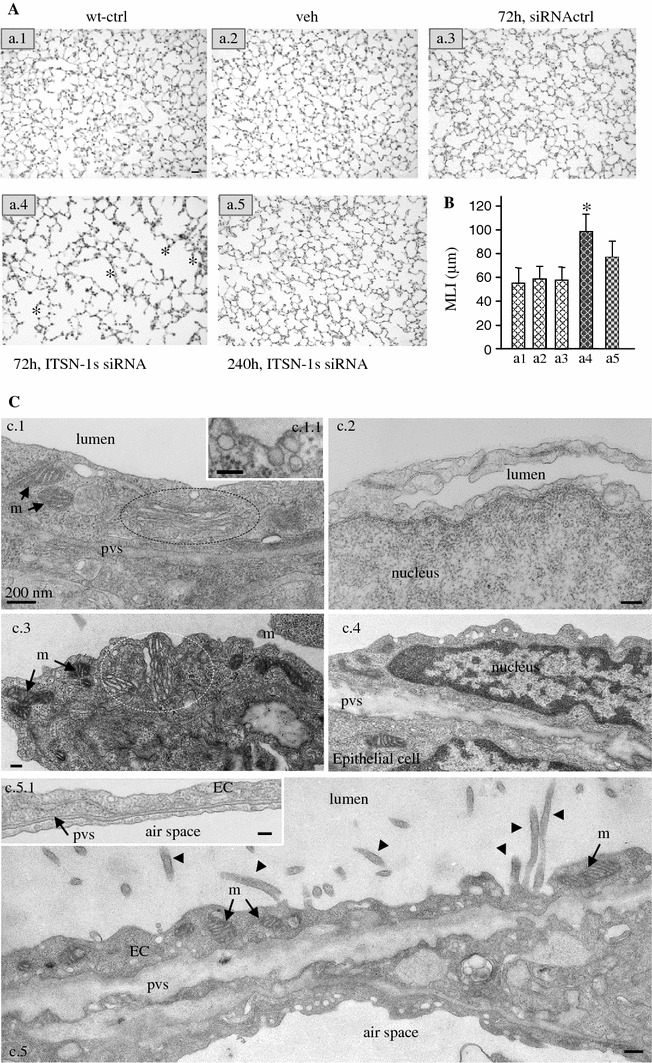
KD_ITSN_ induces lung injury. **A** Representative histo-pathology of mouse lungs from wild-type control (*a1*), vehicle (*a2*), siRNActrl (*a3*), siRNA_ITSN_ (1 single dose) treated mice, at 72 h (*a4*) and 240 h (*a5*) post-siRNA_ITSN_ treatment. H&E staining of paraffin-embedded tissue shows expanded airway spaces (*a4*, *asterisks*) in mouse lungs with acute inhibition of ITSN-1s. *Bar* 60 μm. **B** MLI of control and siRNA_ITSN_-treated mice. All results are representative for at least 3 independent experiments; 30 random power fields were counted from each time point, (*n* = 3 mice/group; *P < 0.05). **C** EM morphological analysis of KD_ITSN_ mouse lungs, 72 h post-siRNA_ITSN_. Fragments of ECs of wild-type mice show normal mitochondria (m), (*a1*, *arrows*), flattened Golgi cisternae, situated in close apposition as stacks (circled area), caveolar profiles open to the lumen or apparently free in the cytosol, (inset *a.1.1*) and a healthy nucleus (*a2*). ECs of KD_ITSN_ show increased number of mitochondrial units (m; *a3*, *a5*), swollen Golgi apparatus (*a3*, circled area) and chromatin condensation (*a4*). Fragment of ECs showing numerous finger-like projections (*a5*, *arrowheads*). Note also the enlarged perivascular space (pvs) and the thick alveolar septum in the KD_ITSN_ by comparison to control (*inset*, *c5.1*). *Bars* 100 nm (*c1.1*); 200 nm (*a1–a5*, *c5.1*). Results are representative for 3 different experiments, with 3 mice/experimental condition

Histological evaluation revealed that the excessive ECs apoptosis caused loss of septal tissue, air space enlargement and alveolar destruction in KD_ITSN_ specimens (Fig. 3A, a4, asterisks). No detectable modifications of pulmonary parenchyma were present in the histology of wild-type, vehicle-, or control siRNA-treated mice (Fig. 3A, a1, a2, a3). The MLI, as a measure of the alveolar size was used to quantify the enlargement of the airspace, as in [31]. At 72 h of KD_ITSN_, MLI was 35 % increased compared to controls (Fig. 3B). Since ITSN-1s protein expression began to recover at 168 h post-siRNA_ITSN_ treatment [19], we also examined the mouse lung histology 3d later (240 h post-siRNA_ITSN_). Less alveolar destruction and limited enlargement of air spaces (Fig. 3A, a5) as well as the decrease in MLI values from 35 to 19.8 % compared to controls (Fig. 3B), suggested that restoring ITSN-1s protein levels re-established pro-survival signaling leading to partial recovery of lung histology. No differences in the extent of ECs apoptosis and lung injury were observed in older mice (24 weeks) versus young mice (6–8 weeks).

Key morphological features of ECs apoptosis and lung injury induced by KD_ITSN_ were also identified by detailed EM surveys of the mouse lungs deficient of ITSN-1s. Frequently, ECs with increased number of mitochondrial units, many of them with abnormal morphology, Golgi apparatus with swollen and unstacked cisternae (Fig. 3C, c3), as well as ECs nuclei displaying evident signs of chromatin condensation (Fig. 3C, c4), were noticed in KD_ITSN_ mouse specimens. For comparison, Fig. 3C, c1, c2 illustrate fragments of wild-type mouse lungs ECs with normal mitochondria, well-organized Golgi, as well as a healthy nucleus. Caveolae, the main vesicular carriers of ECs are numerous and frequently found open to the lumen or apparently free in the cytosol (Fig. 3C, c.1.1). We have often observed in the lungs of KD_ITSN_ mice ECs displaying finger-like protrusions in the lumen of the blood vessels (Fig. 3C, c5, arrowheads), evidence of a dysfunctional activated EC. Recent studies indicate that the membraneous protrusions are very dynamic structures, likely involved in the release of circulating membrane particles, involved in the maintenance and preservation of cellular homeostasis and in promoting defense mechanisms [41, 42]. In addition some of these protrusions are physiologically relevant since they may function in pinocytosis to compensate for deficient endocytic membrane traffic via caveolae and CCVs [19]. Significantly, as a consequence of endothelial injury caused by ITSN-1s deficiency, the permeability of the alveolo-capillary unit was affected, leakage of the protein-rich fluid from the vascular to the interstitial space occurred, causing dilation of the interstitial space at the level of the alveolar septa (Fig. 3C, c4, c5). Overall, we noticed that the lung injury caused by KD_ITSN_ was patchy, mainly localized at the level of the alveolo-capillary units, centered on small vessels (capillaries, post-capillary venules) and around some mid-sized vessels (collecting and muscular venules). Most likely, this patchy distribution reflects the efficient delivery of the siRNA_ITSN_ and thus, downregulation of ITSN-1s. Altogether, the observations indicated that ITSN-1s expression is important for ECs function and survival and that ECs death is an important feature of lung injury in this mouse model.

### Time course of ECs apoptosis in mouse lungs with chronic KD_ITSN_

**Fig. 4:**
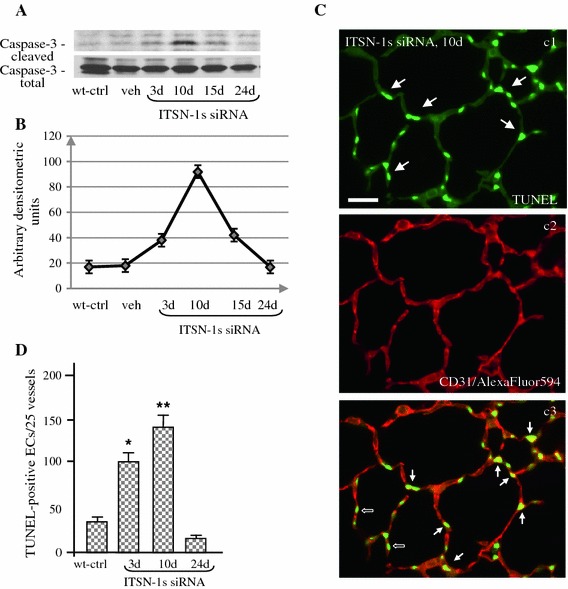
Chronic inhibition of ITSN-1s in mouse lung endothelium causes a peak in caspases-3 activation and significant ECs death at 10d of KD_ITSN_. **A** Western blot analyses of lung lysates (70 μg proteins/lane) of siRNA_ITSN_ and control mice show augmented immunoreactivity for cleaved caspases-3 at 10d of KD_ITSN_, while total caspase-3 expression is unchanged. **b** Densitometric analysis of representative HyBlot CL films indicates more than 3-fold increase in cleaved caspase-3 immunoreactivity at 10d of KD_ITSN_, by reference to untreated mice. Densitometric values ± SEM are representative for three independent experiments. **c** Lung sections of KD_ITSN_ mice (10d) were stained with TUNEL (*c1*) and CD31 Ab/AlexaFluor594 (*c2*) to evaluate the presence of apoptotic ECs within the microvessels of the alveolar wall. The merged image revealed numerous apoptotic ECs (*c3*, *arrows*) as well as epithelial alveolar cells (*c3*, *open arrows*). *Bar* 20 μm. **d** Quantification of TUNEL-positive ECs in medium-sized vessels (50–100 μm) of controls and siRNA_ITSN_-treated mice lungs; *P* < 0.05 for all time points, relative to controls. Results are representative for 3 different experiments, with 3–4 mice/experimental condition

The time course of ECs apoptosis in mouse lungs with chronic KD_ITSN_ (24d post-siRNA_ITSN_), was evaluated by activated caspase-3 immunoreactivity and TUNEL reaction. Western blot applied on total mouse lung lysates revealed cleaved caspase-3 at 3d post-siRNA_ITSN_ delivery (Fig. 4A), with a peak at 10d followed by a gradual decrease to control levels, at 24d. No changes were detected in the total caspase-3 (Fig. 4A). Given the high endothelial content of the lung tissue [30, 39, 43], the lung lysates are a good starting material for biochemical investigations and thus, the findings highly significant for ECs of the lung.

Densitometric analysis indicated more than 3-fold increase in cleaved caspase-3 at 10d, compared to untreated mice (Fig. 4B). TUNEL (Fig. 4C, c1), followed by CD31 Ab staining for specific detection of ECs within the alveolar wall, (Fig. 4C, c2) also revealed increased number of apoptotic ECs nuclei at 10d (Fig. 4C, c3, arrows) compared to 3d KD_ITSN_. We have also detected TUNEL-positive alveolar epithelial cells (Fig. 4C, c3, open arrows). Their apoptotic death can be due to both, (i) the excessive death of ECs and subsequent collapse of the capillary bed, essential for the maintenance of alveolar septa and (ii) a direct effect of KD_ITSN_ at this level. When the extent of ECs apoptosis in medium-sized vessels was evaluated by TUNEL, morphometric analysis indicated that the number of apoptotic ECs reached the highest values at 10d of KD_ITSN_, (Fig. 4D, Table 1), consistent with caspases-3 activation. Moreover, AQP-1 Ab staining and morphometric analysis indicated a further loss of pulmonary microvessels; at 10d, the mean number of lung microvessels per examined area reached the lowest value of 25.6 ± 4, (Table 1). Even with significant apoptosis, no mortality occurred among mice with KD_ITSN_ for 24 consecutive days. Despite continuous and efficient KD_ITSN_, at 24d, the number of apoptotic ECs in medium-sized vessels decreased by 50 % compared to controls and by 85 % compared to KD_ITSN_, at 10d (Table 1), while in the alveolar wall they were detected only occasionally. All together, these observations suggested that extensive ECs death, recorded at 10d of KD_ITSN_, may stimulate ECs hyper-proliferation and perhaps, apoptosis-resistance.

### Chronic KD_ITSN_ mouse lungs show phenotypically-altered ECs

**Fig. 5:**
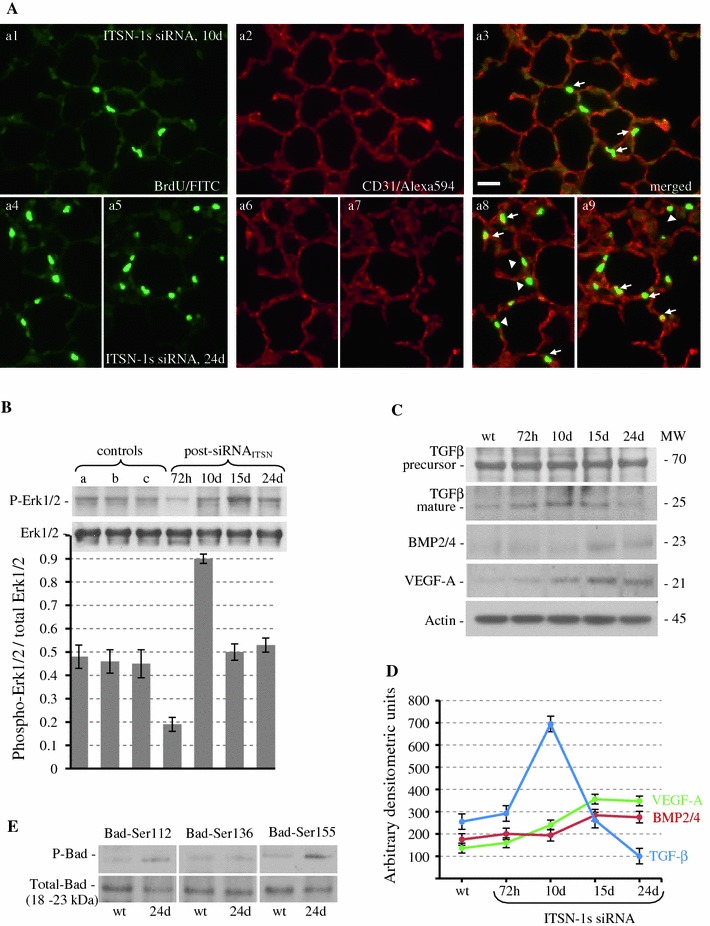
Mouse lung chronically depleted of ITSN-1s show phenotypically-altered ECs. **A** BrdU/FITC Ab immunostaining (*a1*, *a4*, *a5*), followed by CD31/AlexaFluor594 (*a2*, *a6*, *a7*) shows BrdU positive ECs within alveolar wall, at 10d (*a3*, *arrows*) and 24d (*a8*, *a9*, *arrows*). Arrowheads (*a8*, *a9*) point towards BrdU positive alveolar epithelial cells. Bars: 20 μm, (*a1–a9*). **B** Representative Western blots of lung lysates of control, wt-mice (**a**), siRNActrl (**b**) and veh (**c**), 72 h post-injection as well as siRNA_ITSN_-treated mice, at the time points indicated, using phospho-specific Erk1/2^MAPK^ and total Erk1/2^MAPK^ Abs. The graph shows densitometric analysis of Erk1/2^MAPK^ activation (phospho-Erk1/2 relative to total Erk1/2) as mean ± SEM of 3 separate experiments. **C** Expression TGFβ, BMP-2/4 and VEGF-A, in mouse lung lysates of control and siRNA_ITSN_-treated mice, at the time points indicated assessed by Western blot with specific anti-TGFβ, BMP-2/4 and VEGF-A Abs. Actin was used as loading control. D. Densitometric analysis of TGF-β, BMP-2/4 and VEGF-A immunoreactivity. Different Abs and detection conditions did not allow a quantitative assessment of the ratio between the growth factors. E. Evaluation of Bad phosphorylation by Western blotting with anti-phospho Ser^112^-Bad, Ser^136^-Bad and Ser^155^-Bad Abs. The blots were stripped and reprobed with an anti-Bad Ab. The Western blots **C**, **E** are representative for 3–4 different experiments; densitometric analyses **B**, **D** were applied on 3 different HyBlot CL films. Densitometric values ± SEM are representative for 3 independent experiments

The scarce ECs apoptosis detected in chronic KD_ITSN_ mice and the lack of physical evidence of disease in these mice dictated the assessment for compensatory ECs proliferation. We quantified proliferating ECs by BrdU incorporation as described under Methods. Paraffin-embedded lung sections of BrdU-injected mice, controls (wild-type, empty-liposomes and siRNA_ctrl_/liposomes) as well as siRNA_ITSN_-treated mice were incubated with anti-BrdU Ab, followed by CD31Ab, for specific detection of ECs followed by the appropriate fluorophore-conjugated reporters. Acute KD_ITSN_ revealed only sporadic BrdU-positive ECs (not shown). However, at 10d of KD_ITSN_ the BrdU-positive ECs were more numerous (Fig. 5A, a1, a3, arrows) and became greater at 24d of KD_ITSN_, (a4–a9, arrows). Epithelial alveolar cells showed a similar proliferation pattern; BrdU-labeled nuclei were often detected in lung alveoli at 24d of KD_ITSN_, (Fig. 5A, a8, a9, arrowheads). Morphometric analyses applied on medium-sized vessels showed a 1.85-fold increase in BrdU-labeled ECs nuclei at 10d of KD_ITSN_, when apoptosis was at peak and a 2.1-fold increase at 24d, compared to controls (Table 1). The proliferating, BrdU-positive, ECs within alveolar microvessels, normalized per 1.5 × 10^2^ μm of alveolar wall length, increased from 4.4 ± 1.0, in controls, to 8.3 ± 1.5, at 10d to 9.4 ± 1.8 at 24d (Table 1).

The increased cell proliferation in chronic KD_ITSN_ strongly indicated that the survival signaling was restored. Therefore, we evaluated the Erk1/2^MAPK^ activation, known to be affected by ITSN-1s deficiency [8]. Erk1/2^MAPK^ phosphorylation was barely detected at 72 h post-siRNA_ITSN_ delivery (Fig. 5B); however, by 10d, Erk1/2 activation increased about 50 % over the baseline (Fig. 5B, line a), reached a peak at 15d and was still above controls, at 24d of KD_ITSN_; no changes in total Erk1/2^MAPK^ were detected. Control experiments using mice injected with siRNActrl/liposomes complexes (Fig. 5B, line b) and liposomes only (Fig. 5B, line c), 72 h post injection, indicated no significant changes in Erk1/2 phosphorylation compared to the baseline (Fig. 5B, line a). Since Erk1/2^MAPK^ activation can be related to increased expression of growth factors [44], and since the Ras/Erk1/2^MAPK^ is a major signaling pathway downstream of TβR1 [45], we next evaluated the expression of TGF a multi-functional cytokine shown to be involved in ECs proliferation, survival and maintenance of vascular integrity [46]. A modest boost of mature TGFβ at 72–20 % increase compared to control, and a further a 3-fold increase at 10d post-siRNA_ITSN_ treatment, when apoptosis was at peak (Fig. 5C, D), suggested that TGFβ may account for Erk1/2^MAPK^ activation in KD_ITSN_ mice. A gradual decrease to under baseline levels at 24d, were consistent with the stimulatory role of low doses of TGFβ on ECs proliferation [46]. Significant immunoreactivity for the TGFβ homodimeric pro-protein (Mr 74-kDa), was detected at all time points, (Fig. 5C). A dual BMP-2/4 Ab, revealed that the level of mature BMP-2/4, two TGFβ family members able to initiate ECs proliferation [47–49], was increased by 2.2-fold at 15d and 1.7-fold at 24d KD_ITSN_, compared to controls (Fig. 5C, D). Finally, VEGF-A, a multifunctional cytokine that stimulates ECs to survive, proliferate and alter the pattern of their gene expression [50], and whose downstream signaling includes Erk1/2^MAPK^ activation, paralleled BMP-2/4 (Fig. 5C, D). One of likely targets of phosphorylation, by growth factors-activated Erk1/2^MAPK^ is the Bcl-2 family Bad [51, 52]. Bad phosphorylation causes inactivation of its pro-apoptotic properties by blocking the interaction with Bcl-X_L_, [44, 51]. Immunoblotting using site-specific phospho Abs indicated Bad phosphorylation at, Ser^112^ and Ser^155^, (Fig. 5E); No phosphorylation of Bad-Ser^136^, a preferred substrate for PI3K/Akt [53], was identified. PI3K/Akt activation, a downstream target of VEGF signaling [52], was not detected in this cellular context (not shown). We concluded that these multi-functional growth factors may act synergistically to re-establish pro-survival signaling and engage anti-apoptotic pathways, shifting the ECs phenotype toward hyper-proliferation and apoptosis-resistance against ITSN-1s deficiency.

### Chronic KD_ITSN_ induces alveolar and vascular remodeling

**Fig. 6:**
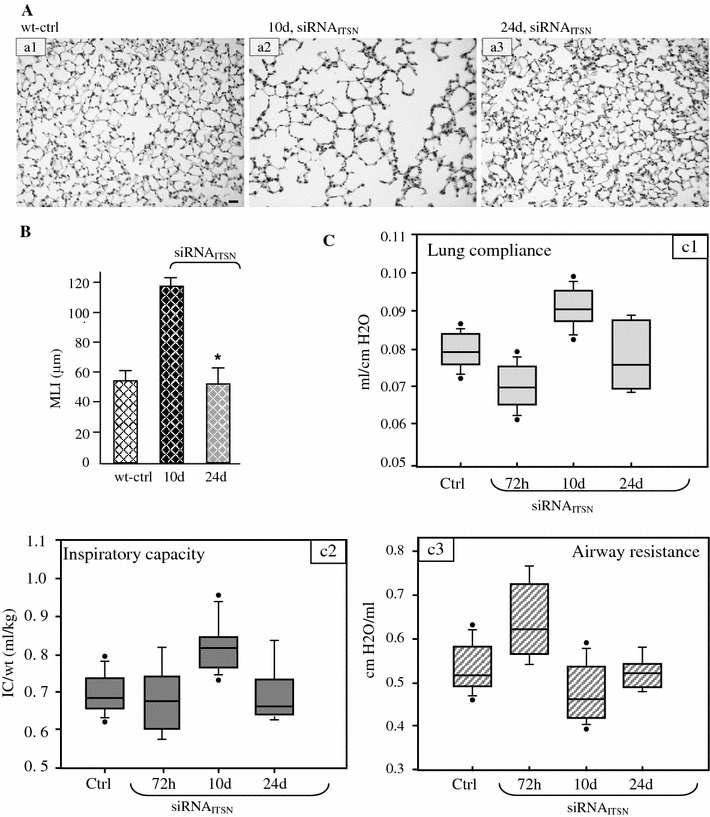
KD_ITSN-1s_ induces mouse lung injury and alters pulmonary function tests. **A** Histology (H&E) of untreated (*a1*), siRNA_ITSN_, 10d, (*a2*) and siRNA_ITSN_, 24d, (*a3*) *Bar* 20 μm. **B** Morphometric analyses of MLI show no difference between the mice with chronic siRNA_ITSN_ (24d) and controls. 30 random high power fields were counted for each group. Data are shown as mean values ± SEM. *P < 0.05 (siRNA_ITSN_ 24d compared to siRNA_ITSN_, 10d). **C** Pulmonary function tests—C_L_, IC and airway R_L_—measurements in KD_ITSN_ mouse. Lines within the boxes show medians; bounds of the boxes show 25th and the 75th percentiles of the data, respectively; the dark circles show outliers. All data are presented as mean ± SEM; *P* < 0.001. Results are representative for three different experiments, with 3–4 mice/experimental condition

H&E staining of mouse lung sections indicated that the alveolar destruction caused by KD_ITSN_ progresses up to 10d of the siRNA_ITSN_ treatment. The lung injury initiated at 3d of KD_ITSN_ is more prominent after 10d of ITSN-1s inhibition (Fig. 6A, a2). Surprisingly, at 24d of KD_ITSN_, the damage is greatly diminished (Fig. 6A, a3); lung morphology, as assessed by H&E staining is similar to controls (Fig. 6A, a1). Apparently, prolonged KD_ITSN_ is followed by endothelial repair and pulmonary architecture remodeling. The lung morphology displays smaller alveoli, densely packed in configuration and few remained signs of tissue destruction. After 10d of KD_ITSN_, the MLI showed a 48 % increase compared to controls and additional 13 % compared to the MLI values for lung specimens, 3d post-siRNA_ITSN_ delivery (Fig. 6B). At 24d of KD_ITSN_, however, despite the extensive apoptotic death and continuous KD_ITSN_, the MLI values were reversed to control levels, consistent with the alveolar remodeling process. In contrast to other vascular remodeling murine models, pulmonary inflammatory infiltrates or vascular enlargements were not detected in KD_ITSN_ mouse lungs.

### Lung cells apoptotic death altered lung mechanics

C_L_, R_L_ and IC in KD_ITSN_ mice were evaluated with Flexivent at 72 h, 10 and 24d after siRNA_ITSN_ treatment. As expected, based on extensive ECs apoptosis and pulmonary edema recorded at 72 h post-siRNA_ITSN_ treatment, C_L_ was decreased by comparison to wild-type mice, when the mean values where compared. (Fig. 6C, c1); IC was not significantly modified (Fig. 6C, c2). At the same time point we found increase in R_L_ (Fig. 6C, c3), most likely due to increased pulmonary recoil, an expected effect of lung fluid accumulation. Interestingly however, at 10d post-siRNA_ITSN_ treatment, the pulmonary function of KD_ITSN_ mice evolved toward increased C_L_, increased IC and low airway R_L_, compared to the wild-type or 72 h post-siRNA mouse lungs. This observation is consistent with the significant and persistent alveolar destruction, enlargement of the air space and increased MLI values. Given the ongoing compensatory cell proliferation, however, the state of endothelium ameliorates and the vascular leakage leading to accumulation of fluid within the interstitial space decreases, as already documented by us [19], fact that may account for C_L_ values at this time point. In keeping with this observation, 24d post-siRNA_ITSN_ delivery, pulmonary function of KD_ITSN_ mice show no notable differences compared to controls.

### KD_ITSN_ mice present increased pulmonary microvessel density and altered Smad activity

**Fig. 7:**
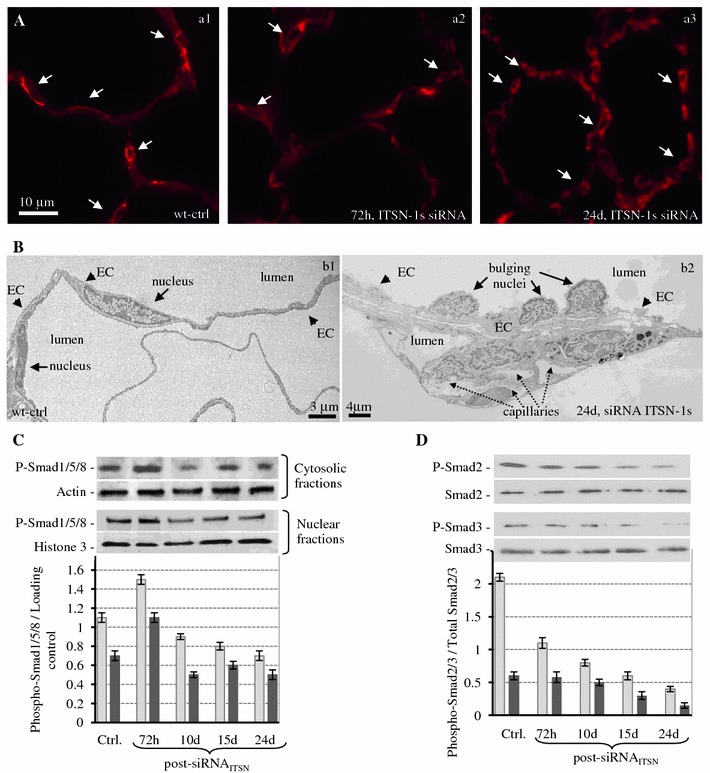
Chronic KD_ITSN-1s_ in mouse lungs induces microvascular remodeling A. Micrographs of GS-1 lectin staining of paraffin-embedded sections show microvessel profiles (*arrows*), within the alveolar walls in control (*a1*), in mice treated with siRNA_ITSN_ for 3d (*a2*) and in mice treated with siRNA_ITSN_ for 24d (*a3*). *Bar* 10 μm. **B** Ultrastructural features of microvascular remodeling in KD_ITSN_ mouse lungs. Two vessels profiles in wt-mouse lung display elongated ECs nuclei (*b1*). Note the relatively uniform thickness of the ECs throughout the vessel perimeter. Segment of a mid-sized vessel in KD_ITSN_ mouse lung shows a distorted endothelium and several nuclei protruding into the lumen (*b2*, *arrows*). New pulmonary microvessels (*dashed arrows*) with narrow openings are abundant and located in very close proximity to each other. **C** Smad1/5/8 phosphorylation in mouse lung cytosolic (light grey bars) and nuclear fractions (dark grey bars) of controls and siRNA_ITSN_-treated mice. Nuclei were isolated from mouse lungs as in [69] and extracts were prepared using NE-PER Nuclear Extraction Reagent (Pierce Biotechnology, Rockford IL). Actin and histone 3 were used as loading controls for the cytosolic and nuclear fractions respectively. **D** Smad2 (light grey bars) and Smad3 (dark grey bars) phosphorylation in total lung lysates of control and siRNA_ITSN_-treated mice. Total Smad2/3 were used as loading controls. Results are representative for 3–5 different experiments

Next, to get insight into the nature of endothelial remodeling caused by KD_ITSN_ we applied GS-1staining, a lectin whose binding is limited to the glycoproteins of the lung microvasculature (< 20 μm diameter), [35]. Fluorescent microscopy analysis showed in controls strong GS-1 labeling of murine lung microvessels and frequent alveolar microvessels profiles (Fig. 7A, a1), consistent with well-vascularized alveolar walls and extensive capillary network. At 10d of KD_ITSN_, GS-1staining was however, weaker and less microvessel profiles were detected within the alveolar wall, consistent with endothelial damage and microvessel loss (Fig. 7A, a2). While the weaker staining may be caused also by technical problems such as accessibility of lectin binding sites within the sections, we believe that this is not the case since similar extent of lack of uniform labeling was noticed throughout the survey of siRNA_ITSN_-treated mice, but not in control specimens. It should also be acknowledged that lack of uniformity in the GS-1s staining may be due to the degree of KD_ITSN_ in different areas of murine lung microvascular bed. In contrast, after 24d of KD_ITSN_ the density of lung alveolar microvessels was increased in comparison not only to 10d KD_ITSN_ but also to wild-type mice (Fig. 7A, a3), suggesting that prolonged KD_ITSN_ triggered microvascular remodeling, characterized by increased number of capillary-sized vessels. Quantitative measurements, (Table 1), indicated that the mean number of lung microvessels per 30,000 μm^2^ surface area in chronic inhibition mice, at 24d reaches 443 ± 7.1, ~ 20 % higher than control levels (365 ± 9). Evidence of EC proliferation and microvascular remodeling was further obtained by EM morphological analyses of KD_ITSN_ mouse lungs, (Fig. 7B). While under control conditions, ECs lining the mouse lung vessels show a relatively uniform diameter/thickness and typical, elongated nuclei (Fig. 7B, b1), in KD_ITSN_ specimens we have detected vessels profiles where ECs were distorted, varied in their thickness and displayed several EC nuclei protruding into the lumen, suggestive of endothelial hyper-proliferation occurring at this level (Fig. 7B, b2). Luminaly bulging ECs nuclei is a structural feature of forming and developing blood capillaries, previously documented in other tissues and systems (Manzke, E., 2005). Note also the underlying area rich in growing vessels (Fig. 7B, b2, dashed arrows) with small, narrow lumina and very close to each other, suggestive of an ongoing remodeling process.

Downstream of TGFβ and BMP-2/4 signaling are Smad proteins, involved in ECs proliferation and angiogenesis [46]. First, Smad1/5/8 activity was evaluated by Western blot analyses of both cytosol and nuclear fractions prepared from lung tissue of control and KD_ITSN_ mice at 3, 10, 15 and 24d post-siRNA_ITSN_ delivery, using a phospho-Smad1/5/8 Ab, (Fig. 7C). Phosphorylation of Smad1/5/8/, downstream events of both ALK1, a TβR1 whose expression is restricted to ECs, and BMPR2 [46] was slightly increased in both fractions at 72 h post-siRNA_ITSN_ treatment; then a gradual decrease, about 30 % lower compared to controls and 72 h post-siRNA_ITSN_, was detected. Since previous reports demonstrated that TβR1 signaling is not directly affected by impaired endocytosis [54], and that inhibition of BMPR2 endocytosis does not affect Smad1/5/8 phosphorylation, but affects their nuclear translocation [55], it is likely that in a cellular environment deficient in caveolae and CCVs endocytosis, the BMPR2 endocytic traffic is altered due to upregulation of compensatory alternative endocytic pathways, [19]. Both Smad1/5/8 phosphorylation and nuclear translocation still occur, but less efficiently compared to controls. At 72 h post-siRNA_ITSN_, the alternative endocytic pathways do not function efficiently enough (Predescu, 2012) to affect BMPR2 endocytic internalization; the receptor is still on cell surface and less internalized due to caveolae and CCVs impaired endocytosis, and thus its signaling may be enhanced, resulting in increased Smad1/5/8 phosphorylation and nuclear translocation, at this time point.

Moreover, the Smad2/3, downstream of TβR1 show decreased levels of phosphorylation, Fig. 7D. The levels of Smad2/3 are unchanged, suggesting that Smad2 and Smad3 are not targeted for degradation. It appears that the typical TβR1/ALK5 signaling has been shifted from the Smad2/3 activation toward a less common Ras/Erk1/2^MAPK^ pathway, with protective effects on ECs and lung vasculature. Significantly, as already reported, Smad2/3 inhibition is consistent with the ECs proliferation [46].

To rule out a possible involvement of Smad 4 and inhibitory Smad 7 in the inhibition of Smad2/3 activity, we analyzed by Western blot the expression of the two Smad proteins in mouse lung lysates (Online Resource 1). No changes in the expression of the common Smad 4 were detected. The levels of the inhibitory Smad7, display a gradual decrease, reaching a 30 % decrease at 24d post-siRNA_ITSN_ delivery compared to Smad7 in the lung lysates of wt-mice. The observations further support the concept that the impaired endocytic traffic of TβR1 and activation of Erk1/2^MAPK^ account for Smad2/3 inhibition.

**Fig. 8:**
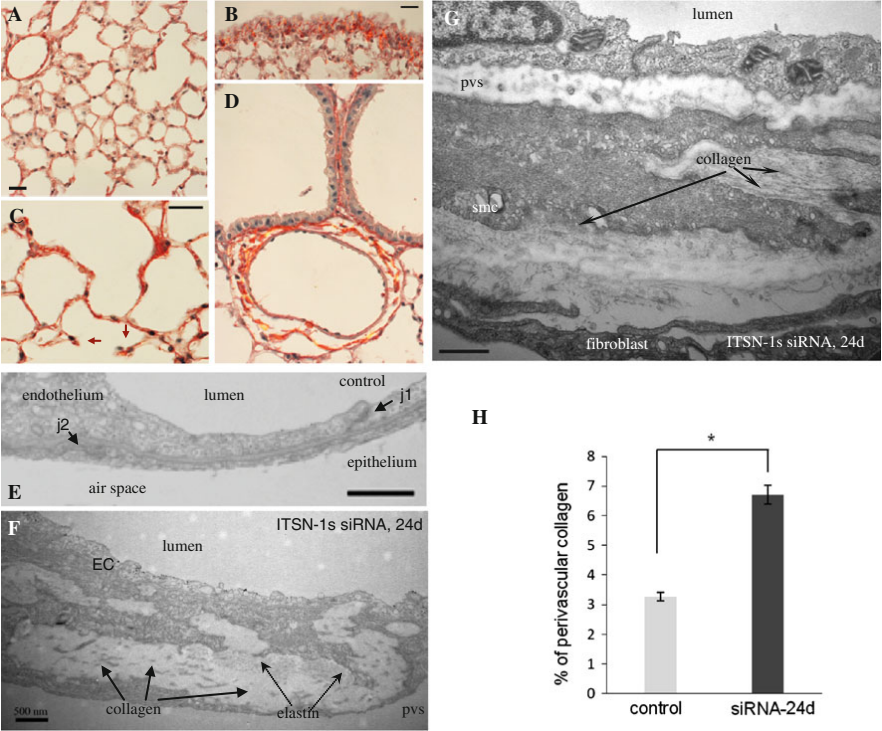
Chronic ITSN-1s deficiency leads to dense perivascular collagen depositions Representative micrographs of Picrosirius Red-stained lung sections of control (**A**) and siRNA_ITSN_-treated mice for 24d (**B–D**), to assess collagen deposition. Short septal walls remnants were often noticed with collagen bundles at the tip (**C**, *arrows*). *Bars* 20 μm. Electron micrographs show segments of the alveolar septal wall in a control microvessel (**E**), as well as in a postcapillary venule (10–15 μm diameter) (**F**), and a precapillary arteriole (20–25 μm diameter) (**G**) of ITSN-1s deficient mice. Collagen fibrils (**G**, *arrows*), and fibrilar material (proteoglicans, elastic fibers, etc.), accumulated in the basement membrane between the ECs, smc and fibroblast. j1—interendothelial junction, j2—epithelial junction, EC—endothelial cell, pvs—perivascular space, smc—smooth muscle cell. *Bar* 500 nm. **H** Quantification of the amount of collagen encircling middle-sized lung vessels in control and ITSN-1s chronic inhibition mice. Results are representative for 3 different experiments, with 3 mice/experimental condition. Collagen layers were measured in 20–25 randomly chosen high power fields comprising 25 medium-sized blood vessels for each experimental condition. Data are expressed as mean ± SEM, *P < 0.05

Since Erk1/2-dependent, Smad2/3-independent TGFβ signaling has been related to collagen production [56, 57], we further address the lung remodeling and try to elucidate the mechanism behind the reduction in C_L_ in KD_ITSN_ mice (24d). To this intent, we evaluated the status of extracellular matrix deposition, with focus on collagen. Chronic ITSN-1s deficiency leads to collagen depositions at the level of subpleural space (Fig. 8B), in the alveolar septa (Fig. 8C) as well as in the perivascular spaces (Fig. 8D). Short septal walls remnants were often noticed with collagen bundles at the tip (Fig. 8C, arrows). EM studies further indicate that collagen fibrils (F, arrows), and fibrilar material (proteoglycans, elastic fibers, etc.), accumulated in the basement membrane between the ECs, smooth muscle cells and fibroblasts. We estimated that on 23,250 μm^2^, (the average surface of selected area), the percentage area occupied by collagen represents only 3.26 ± 0.14 % in controls, while in chronic KD_ITSN_ mice, (24d), reaches 6.71 ± 0.31 %, Fig. 8H.

## Discussion

In this study we evaluated the in vivo effects of ITSN-1s deficiency on pulmonary ECs apoptosis and lung homeostasis using a KD_ITSN_ mouse model in which ITSN-1s was efficiently and specifically inhibited for 24 consecutive days, via i.v. delivery of siRNA_ITSN_/cationic liposomes. Acute KD_ITSN_ and loss of Erk1/2^MAPK^ survival signaling caused significant ECs apoptosis leading to endothelial damage, microvessels loss and alveolar destruction. These results are in keeping with our previous studies showing that ITSN-1s deficiency interferes with ECs function and survival by inhibition of MEK/Erk1/2 phosphorylation [8]. Increased ECs death as revealed by TUNEL and caspase-3 activation began at 3d and peaked at 10d of KD_ITSN_. As a consequence, lung injury occurred rapidly, allowing us for a detailed time course analysis of structural destruction and disease progression.

Studies have shown that in the lungs of chronically hypoxic rats treated with the VEGFR-2 inhibitor SU5416, pulmonary ECs death preceded development of severe PAH, associated with precapillary arterial occlusion by proliferating ECs [14]. Mouse lung ECs death, as caused by KD_ITSN_, was a prerequisite for the following ECs proliferation, repair and remodeling process. Within days, these normally quiescent cells with a low turnover rate, began to hyper-proliferate, indicating that the survival signaling, lost due to KD_ITSN_, was re-established.

**Fig. 9:**
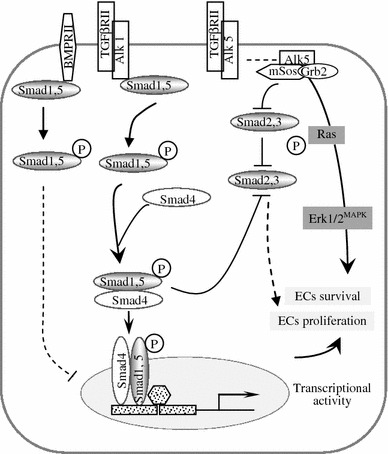
Schematic representation of TGFβ-Smads/Erk1/2^MAPK^ signaling switch in ECs of KD_ITSN_ TGFβ transmits its signal by binding to type II and type I receptors, resulting in activation of type I receptor and subsequent phosphorylation of R-Smads. ITSN-1s binds mSos [7] and thus, KD_ITSN_ may increase mSos availability for Grb2 interaction, and preferential formation of ALK5/mSos/Grb2 signaling complex. This may result in ineffective assembly of ALK5/Smad2/SARA complexes and subsequent alteration of the Smad2/3-Erk1/2 signaling balance toward persistent Ras/MEK/Erk1/2 activation. ALK5 functions to activate Ras/Erk1/2^MAPK^ necessary for restoring pro-survival signaling, lost due to KD_ITSN_. This signaling event may suppress Smad2/3 phosphorylation. Note that Smad2/3 phosphorylation can be inhibited not only upon Ras/Erk1/2 activation but also upon Smad1/5/8 activation. Smad1/5/8 phosphorylation downstream of BMPR2 is triggered while the receptor is at the plasma membrane; the transcriptional response is however, dependent on BMPR2 internalization [55]

Expression of TGFβ a cytokine that regulates diverse and often contradictory functions, in a milieu- and cell type-dependent manner, [46] was increased. In a cellular environment deficient in Erk1/2 activity and with altered endocytic trafficking [19] caused by ITSN-1s deficiency, the typical TβR1/ALK5 signaling has been shifted from Smad2/3 activation toward a less common Ras/Erk1/2^MAPK^ pathway, with protective effects on ECs and lung vasculature. The mechanisms underlying TGFβ/ALK5-dependent Ras/Erk1/2 activation is an unresolved issue of considerable interest due to its established role in providing a growth advantage [58]. TGFβ induces Ras/Erk signaling through direct phosphorylation of the adaptor protein SchA, [59] leading to its association with Son of Sevenless (Sos), a Ras GTP/GDP exchange factor, and the growth factor receptor bound protein 2 [60]. Notably, ITSN-1s associates with Sos in a protein complex that excludes Grb2 [7], raising the possibility that KD_ITSN_ may increase Sos availability for Grb2 interaction, and thus, preferential formation of ALK5/Sos/Grb2 signaling complex. This may result in ineffective assembly of ALK5/Smad2/SARA complexes and subsequent alteration of the Smad2/3-Erk1/2 signaling balance toward persistent Ras/MEK/Erk1/2 activation. In addition, while Smad1/5 phosphorylation, downstream of BMPR2 occurs at the plasma membrane and is not affected by deficient endocytic trafficking, continuation of Smad signaling requires receptor internalization [55], which cannot occur efficiently due to impaired endocytic traffic caused by KD_ITSN_ [2, 19]. Apparently, the endocytic dysfunction and loss of ITSN-1s-mediated Erk1/2^MAPK^ activation, directed Smad1/5/8 activity, downstream of BMPR2 and ALK1, to avoid excessive ECs proliferation, while facilitating ECs proliferation and the recovery of vessel loss (Fig. 9). Moreover, despite the TβR1/ALK5 ability to signal without endocytic internalization, events such as Smad1/5/8 and Ras/Erk1/2^MAPK^ activation may have an inhibitory effect on Smad2/3 pathway in lung ECs impaired in endocytic trafficking [61].

Sustained activation of Erk1/2 is necessary for cells progressing from G1 to S-phase and it is usually associated with induction of D-type cyclins and assembly with their catalytic partners that phosphorylate the retinoblastoma proteins resulting in release of E2F and transcription of genes required for DNA replication [10]. Thus, Erk1/2-Smad2/3 signaling switch may affect two well-known molecular mechanisms of cell growth arrest by TGFβ in the G1-phase of cell cycle, (i) downregulation of D type cyclins and cyclin-dependent kinases expression [62–64] and (ii) activation of cyclin-dependent kinases inhibitors [65], events that may account for initiation of the ECs hyper-proliferative phenotype.

Bad phosphorylation by growth factors activated Erk1/2^MAPK^, and inactivation of its pro-apoptotic properties, already demonstrated in cultured neurons and in models of cerebral ischemia, [66] may be part of the molecular mechanism leading to the EC apoptotic-resistant phenotype caused by chronic KD_ITSN_ and lung repair.

Although the crosstalk between the TGFβ and other signaling pathways was not addressed in detail in these experimental settings, the studies described here demonstrate that Erk1/2^MAPK^ activation, Bad phosphorylation and the perturbed Smad signaling downstream of ALK5 and BMPR2, play an important role in lung ECs survival and injury repair.

Vascular remodeling induced by chronic KD_ITSN_ was assessed by a detailed survey of AQP-1 and GS-1 lectin labeling, widely used to visualize the mouse lung microvessels [35]. Excessive apoptotic ECs death recorded at 72 h and 10d post-siRNA treatment, and the decrease of microvessels density were followed by ECs proliferation and microvascular repair/development process that not only restored the number of lung microvessels, but apparently, added new microvessels to the system. Increased expression of both VEGF-A, a central growth and survival factor for the ECs [50] and BMP2/4 already implicated in capillary sprouting via Erk1/2^MAPK^ signaling [49], may be, at least partly responsible for this KD_ITSN_ mouse phenotype. Moreover, the apoptotic/proliferative ECs coexistence (high cellular death/growth) in the murine lung endothelium at 10d of KD_ITSN_ indicates the conversion time of normal, surviving ECs to a hyper-proliferative phenotype. What initiated out of necessity for tissue replenishment, progresses to microvascular remodeling due to a newly acquired hyper-proliferative ECs phenotype. As shown KD_ITSN_ in the mouse lungs also affected lung epithelial cells—either extensive ECs apoptosis or a direct siRNA effect on lung epithelium may account for the observed epithelial cells apoptotic death. Lung parenchymal proliferation might be expected as a result of increased number of blood vessels, providing the energy substrate for accelerated alveolar replenishment [67]. Thus, it is not surprising that BrdU staining of lungs sections with chronic KD_ITSN_ revealed also a multitude of proliferative epithelial cells. At the end of the study no significant difference was found between MLI of these specimens and controls. Even if the MLI shows overall equality, lung morphology after 24d of ITSN-1s suppression did not resemble controls. Few increased airspace regions were in complementary distribution to various areas with closely packed alveoli, smaller in size than in controls. This data suggest that hyper-proliferative pulmonary ECs prompted pneumocytes proliferation, reactive to the favorable milieu created by the vascular development. While the role of epithelial cell apoptosis in lung repair and remodeling cannot be disregarded, given the efficient and specific delivery of siRNA_ITSN_/cationic liposomes to pulmonary ECs and the high endothelial content of the lung tissue [30], the findings reported here are highly relevant for ECs of the lung.

During the 24d of ITSN-1s deficiency, pulmonary function of KD_ITSN_ mice evolved from low C_L_ and increased R_L_ at 72 h post-siRNA delivery, toward increased C_L_ and decreased R_L_ at 10d, while, at the end of the study both parameters were close to control values. Compensatory proliferation, observed at 10d post-siRNA treatment, contributed to the amelioration of endothelial injury and reduced the pulmonary fluid buildup, events that are behind the change in pulmonary mechanics at 10d. Depositions of matrix components (collagen and elastin fibers) and increase in interstitial cell population were commonly seen at the ultrastructural level in mouse lung vessels with chronic KD_ITSN_. The remodeling of the alveolo-capillary functional unit (i.e. epithelial cells proliferation, collagen deposition within alveolar septum, increased microvessels density) further improved the C_L_, R_L_ and IC of the injured lungs.

Taken together, our data showed that in the pulmonary tissues of mice deficient of ITSN-1s, impaired endocytic membrane traffic and initial ECs apoptosis was followed by ECs phenotypic changes toward hyper-proliferation and apoptosis-resistance against ITSN-1s deficiency as well as microvascular remodeling in the remaining pulmonary microvascular bed. Interestingly, TβR1 downstream signaling was apparently switched from the canonical Smad2/Smad3 toward Erk1/2^MAPK^. This signaling switch proved to be central for ECs survival, maintaining vascular integrity and lung homeostasis.

The findings are relevant considering that ITSN-1s is a general endocytic protein, a regulator of mitochondrial apoptosis and a GrB substrate [15]. Under inflammatory conditions and increased GrB levels, loss of full-length ITSN-1s may cause endocytic dysfunction, loss of pro-survival signaling and apoptotic ECs death. As result, loss of blood vessels, damage of the alveolar wall, abnormal or incomplete tissue repair may occur. These events are major determinants able to cause during disease progression the selection of hyper-proliferative and apoptotic-resistant cell phenotypes [68]. The findings implicate ITSN-1s, a key endocytic and pro-survival protein of lung endothelium, and the biological processes regulated by it (i.e., endocytosis, transcytosis, cell survival and apoptosis) in vascular remodeling and pathology of lung disorders.

## Electronic supplementary material

Below is the link to the electronic supplementary material.

Supplementary material 1 (DOCX 143 kb)
