# Supplementary material for: In vivo knockdown of intersectin-1s alters endothelial cell phenotype and causes microvascular remodeling in the mouse lungs
Source: Apoptosis. 2012 Oct 7;18(1):57–76. doi: 10.1007/s10495-012-0762-x (PMC3543613; doi:10.1007/s10495-012-0762-x)
Supplement: Supplementary file 1 — Supplementary material 1 (DOCX 143 kb) [file 10495_2012_762_MOESM1_ESM.docx]

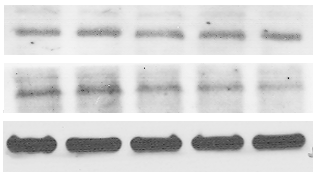


**Online Resource 1.** Smad4 and Smad7 protein expression in total lung lysates of control and siRNA_ITSN_-treated mice. Actin was used as loading control. The graph shows densitometric analyses of Smad4 (light grey bars) and Smad7 (dark grey bars) immunoreactivity. Densitometric values ± SEM are representative for 3 independent experiments.

experiments.

Smad7 -

Smad4 -

Actin -
